# Comparative analysis of retinal photoplethysmographic spatial maps and thickness of retinal nerve fiber layer

**DOI:** 10.1371/journal.pone.0284743

**Published:** 2023-05-05

**Authors:** Jan Odstrcilik, Radim Kolar, Folkert Horn, Ralf-Peter Tornow

**Affiliations:** 1 Department of Biomedical Engineering, Faculty of Electrical Engineering and Communication, Brno University of Technology, Brno, Czech Republic; 2 Department of Ophthalmology, Friedrich-Alexander-University of Erlangen-Nuremberg, Erlangen, Germany; Tsukazaki Hospital, JAPAN

## Abstract

The paper presents a comparative study of the pulsatile attenuation amplitude (PAA) within the optic nerve head (ONH) at four different areas calculated from retinal video sequences and its relevance to the retinal nerve fiber layer thickness (RNFL) changes in normal subjects and patients with different stages of glaucoma. The proposed methodology utilizes processing of retinal video sequences acquired by a novel video ophthalmoscope. The PAA parameter measures the amplitude of heartbeat-modulated light attenuation in retinal tissue. Correlation analysis between PAA and RNFL is performed in vessel-free locations of the peripapillary region with the proposed evaluating patterns: 360° circular area, temporal semi-circle, nasal semi-circle. For comparison, the full ONH area is also included. Various positions and sizes of evaluating patterns in peripapillary region were tested which resulted in different outputs of correlation analysis. The results show significant correlation between PAA and RNFL thickness calculated in proposed areas. The highest correlation coefficient *R*_temp_ = 0.557 (p<0.001) reflects the highest PAA-RNFL correspondence in the temporal semi-circular area, compared to the lowest value in the nasal semi-circular area (*R*_nasal_ = 0.332, p<0.001). Furthermore, the results indicate the most relevant approach to calculate PAA from the acquired video sequences is using a thin annulus near the ONH center. Finally, the paper shows the proposed photoplethysmographic principle based on innovative video ophthalmoscope can be used to analyze changes in retinal perfusion in peripapillary area and can be potentially used to assess progression of the RNFL deterioration.

## Introduction

Glaucoma is classified as optic neuropathy characterized by a progressive loss of ganglion cells and their corresponding axons, that is, nerve fibers. This results in a decrease of the retinal nerve fiber layer (RNFL) thickness and leads to visual field defects [1]. RNFL thickness is typically assessed by optical coherence tomography (OCT), which has become a standard tool in many clinical settings in ophthalmology [1]. Furthermore, OCT angiography (OCTA) has been introduced as a noninvasive and reproducible method to provide a reliable evaluation of the retinal microvasculature [2]. There has been a high effort to reveal the correspondence between the retinal microvasculature and RNFL using OCT / OCTA in the last decade. Many studies [3–7] have been published on the topic of correlation between optic nerve head (ONH) and peripapillary blood vessel density (assessed by OCTA) and other glaucoma diagnostic parameters (RNFL thickness, visual field parameters, etc.). Generally, the published results show reduced ONH blood vessels and retinal perfusion associated with structural and functional glaucomatous damage.

Despite the many benefits of OCT(A), it is still a rather expensive and unavailable instrument, and also needs an experienced clinical evaluator. In addition, an examination typically takes several minutes, which is not very convenient for screening purposes. Nevertheless, a relatively new imaging modality, called Laser speckle flowgraphy (LSF), can be also used for assessment of the retinal microvascular blood flow. The LSF is based on changed laser speckle statistics for moving targets (mainly red blood cells), which can be evaluated by computation of image contrast from the acquired video data. The primary outcome of this approach is called the mean blur rate (MBR), a parameter that depends on the velocity [8]. Yokoyama, et al. [9] have shown a reduction in ONH microcirculation (assessed by MBR) in glaucomatous eye with RNFL deterioration. Similarly, Shiga et al. [10] found that LSF is a useful technique to monitor the severity of glaucoma, by measuring ONH perfusion (again assessed using MBR).

Generally, it can be concluded that recently published studies on the above-mentioned modalities have shown a significant reduction of underlying blood flow in ONH and peripapillary area of the glaucomatous eye with ongoing structural changes. The decrease in blood vessel density and blood flow in glaucomatous eyes suggests also an application of well-known photoplethysmographic (PPG) principle for assessment of ONH and peripapillary perfusion changes. The authors of this study have already developed an experimental video-ophthalmoscope (VO), which enables the application of the PPG principles to retinal tissue [11]. This device has been designed as a lightweight and cost-effective solution for the easy acquisition of retinal video sequences. Due to the unique design, i.e. simplified illumination and acquisition part in comparison to standard fundus camera, it enables to acquire binocular retinal video sequences simultaneously too. Then, the authors have proposed a unique image processing pipeline to perform basic quantitative retinal PPG for evaluation of retinal perfusion [11, 12]. Currently, the authors have published a robust approach for retinal video PPG analysis utilizing the Fourier transformation [13]. In this paper, we summarize our previously published processing steps and further extend evaluation of the proposed perfusion-related parameter—the pulsatile attenuation amplitude (PAA) with respect to RNFL thickness measured by standardized clinical OCT. The clinical relevance of PAA has been already shown in [12, 13]. In this paper, we propose different evaluation patterns for PAA calculation to find maximum spatial relation (in a term of correlation) between PAA and RNFL. However, ongoing research in this field has further objectives to transfer the results into the clinical space. Therefore, we further evaluate the diagnostic potential of our PPG principle (i.e. PAA parameter) with respect to glaucoma diseases and consequent structural changes in RNFL.

## Methods

### Data acquisition and experimental dataset

#### Acquisition device

The retinal video sequences were acquired using our recently developed binocular video-ophthalmoscope (VO) [11]. The VO utilizes the principle of standard fundus cameras. An aerial image of the retina in a focal image plane is produced by an ophthalmic lens. The focal image plane contains a field stop to restrict the field of view to 20°×15° centered to the ONH and a fixation target with small OLED display to maintain stable fixation of examining subject during data acquisition. The illuminating light has a wavelength equal to 577 nm (LED), which corresponds to high absorption of light by blood and thus enables to measure intensity modulated by blood volume changes during the cardiac cycles. The device utilizes a CMOS camera (UI-3060 Rev 2, USB 3.0, IDS Imaging Development Systems GmbH, Germany) with the frame rate 25 fps. Due to the binocular setup, two video sequences (left and right eye) can be measured simultaneously during a single session. In this study, we analyze the sequences of both eyes independently. Nevertheless, simultaneous acquisition offers further possibility to analyze and evaluate inter-eye time shifts and related parameters in the future.

#### Data acquisition and subjects

A total number of 136 video sequences from 136 eyes of 70 subjects (67 left and 69 right eyes, respectively) were acquired. The data were measured during the study under the Erlangen Glaucoma Registry (EGR) clinical trial (www.clinicaltrials.gov, NCT00494923). The Erlangen Glaucoma Registry is a clinical registry for cross-sectional and longitudinal observation of patients with open angle glaucoma (OAG) or glaucoma suspect. All control subjects and patients were thoroughly examined by slit-lamp inspection, applanation tonometry, fundoscopy, gonioscopy, standard automated perimetry (SAP), and papillometry. Optic disc evaluations of patients and controls were based on 15-degree color images (Zeiss telecentric fundus camera, Carl Zeiss, Meditec). All eyes included in the study had clear optic media. On the day of the examination, the intraocular pressure was equal to or less than 22 mmHg. Exclusion criteria were all eye diseases other than glaucoma, diabetes mellitus and color vision anomalies. More detailed information about this glaucoma study, all applied examinations and the classification of the subjects based on these examinations can be found in our previously published clinical paper [12]. All participants signed an informed consent form approved by the Ethics Committee of the Friedrich-Alexander University of Erlangen-Nürnberg, in accordance with the Declaration of Helsinki. The written consents of the participants are archived at the University. The subjects were equally divided into males and females. Mean age of subjects was 67±11 years. The refraction values of subjects were in the range -5.6 to 6.4 (spherical equivalent).

Based on the clinical results, the eyes were divided into four groups (G_norm_, G_OHT_, G_pre_, G_per_) according to the stage of glaucomatous changes. The G_norm_ group includes 21 eyes without any signs of eye diseases and normal status of RNFL. G_OHT_ includes 25 eyes diagnosed with ocular hypertension (OHT). The subjects had earlier intraocular pressures above 21 mmHg upon repeated measurements. They had normal ONHs and they were without any signs of RNFL deterioration. All of them had a ‘non-perimetric’ visual field result with white-on-white perimetry. G_pre_ consists of 22 eyes with pre-perimetric glaucoma. They showed glaucomatous abnormalities of the ONH and localized or diffuse loss of RNFL. Computerized visual field examinations with white-on-white perimetry were normal. G_per_ includes 68 eyes with primary open-angle glaucoma. All subjects of this ‘perimetric’ glaucoma group had glaucomatous ONH damage and local and/or diffuse visual field loss in white-on-white perimetry.

**Fig 1:**
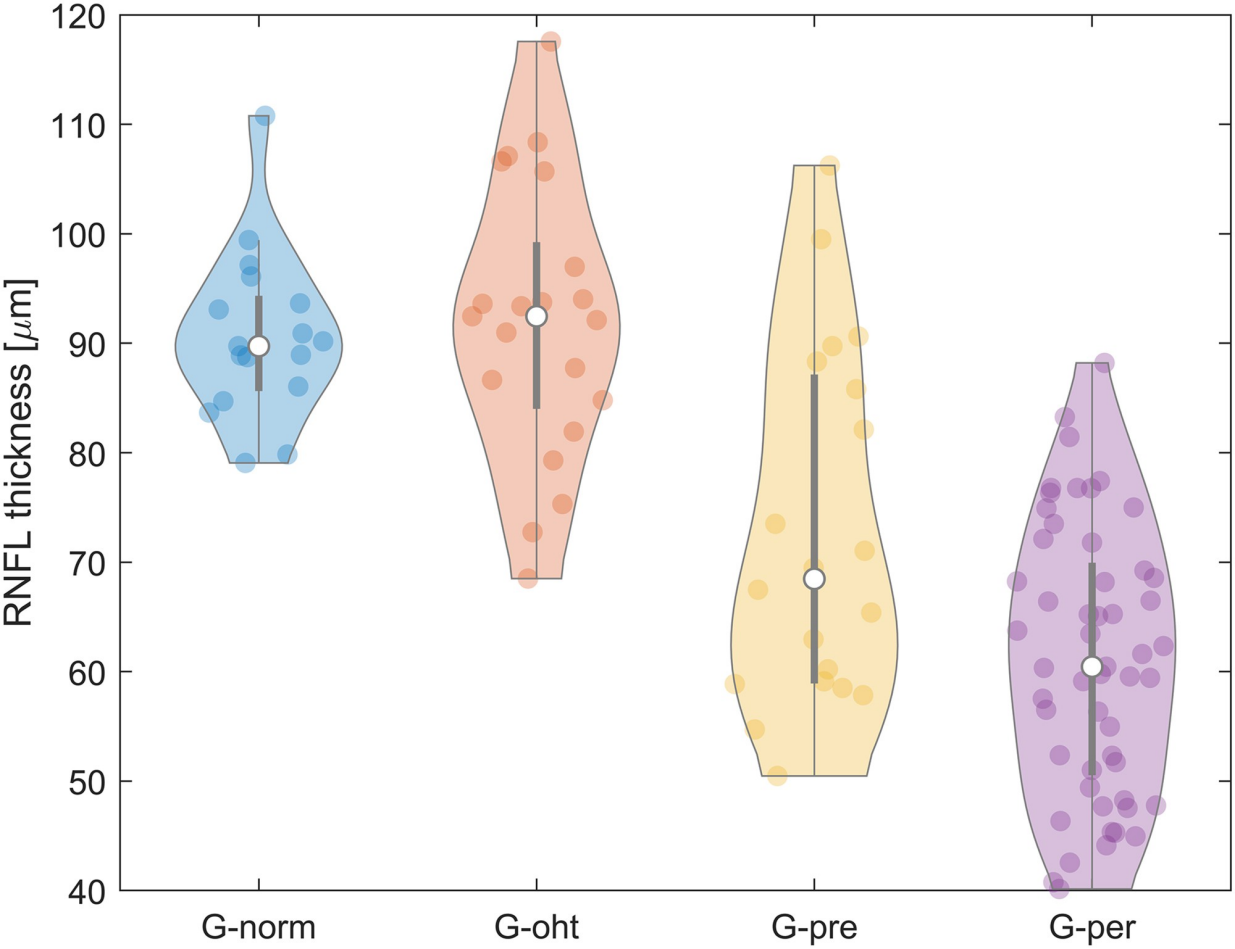
A violin plot of RNFL thickness distribution of G_norm_—normal eyes, G_OHT_—OHT eyes, G_pre_—pre-perimetric glaucoma, G_per_—perimetric glaucoma. The white dot represents the median value of the RNFL thickness in each group, the thick gray bar in the center represents the interquartile range, while the thin gray line shows the rest of the distribution, except outliers. On each side of the gray line is a kernel density estimation to show the histogram shape of the RNFL data. Wider sections of the violin plot represent a higher histogram probability value and the thinner parts represent a lower probability.

Before the acquisition of the video sequences, all subjects had 10 minutes rest time in the examination room before measurement to reduce the possible influence of physical exertion. After aligning the instrument with the subject’s eye (with dilated pupils) using a head and chin rest and an adapted XYZ mount of a slit lamp, video sequences were acquired for each subject with a duration of 10 seconds each (with frame rate of 25 fps). Registered video sequences were used to generate mean images by averaging the particular video frames. Before further analysis, the mean images were used for subjective evaluation of the quality and usability of particular video data. Low quality video sequences due to blurriness were excluded from the study. Furthermore, sequences where the ONH was not located in the center of the field of view (i.e. ONH was positioned too close to the image border), were removed as well. Finally, the original clinical trial dataset of 136 video sequences was reduced to 111 video sequences (17 eyes in G_norm_, 21 eyes in G_OHT_, 20 eyes in G_pre_, 53 eyes in G_per_). All subjects included in the study had a circular OCT scan (3.5 mm diameter) centered at the ONH using the Spectralis OCT (Heidelberg Engineering, Germany) to measure the RNFL thickness. The OCT scans were acquired in the high-speed circular mode (standard procedure in EGR clinical trial) with ART (Automatic Real-time Tracking) function activated. The signal strength and signal quality of acquired data were controlled during the acquisition so that the software could measure thickness of RNFL with high precision. The mean RNFL thickness of all subjects included in the analysis is 73.53±18.33 μm; divided into the glaucoma groups: 90.64±7.65 μm (G_norm_), 91.88±12.55 μm (G_OHT_), 72.60±16.09 μm (G_pre_), 61.13±12.20 μm (G_per_). Fig 1 shows distribution of the RNFL thickness in the analyzed dataset.

### Data preprocessing

#### Registration of video sequences

**Fig 2:**
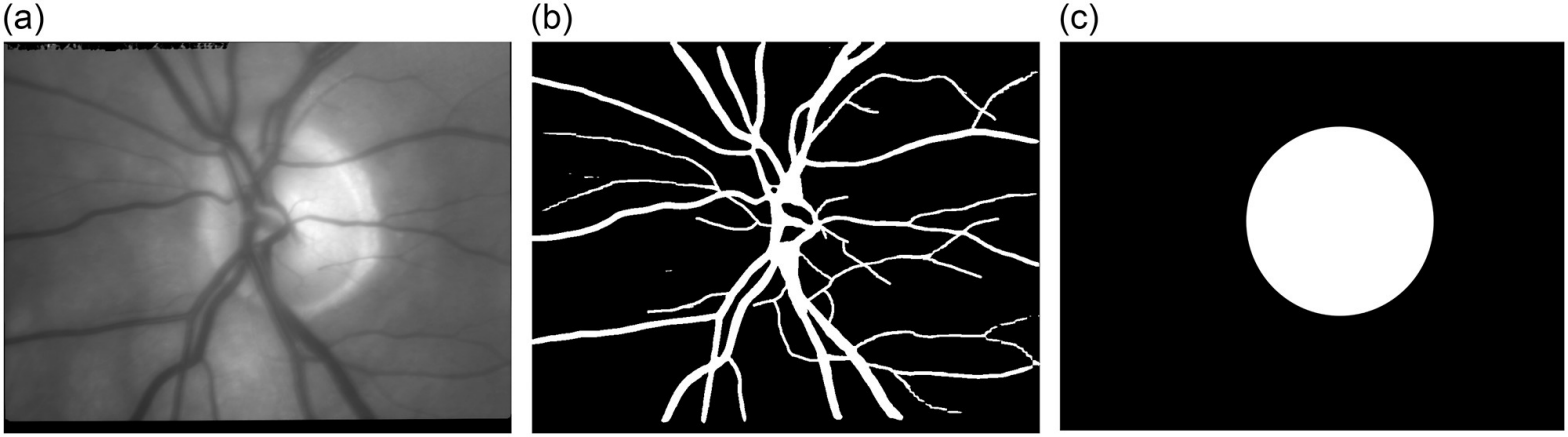
Data preprocessing steps. a) an original mean image calculated from a video sequence after frame-to-frame registration, b) binary mask of segmented blood vessels, c) binary mask of ONH.

Acquired video sequences were registered to compensate for eye shifts and rotation using a fast two-stage process. The phase correlation in the first stage removes large eye movements, while the Lucas-Kanade tracking approach with adaptive selection of tracking points removes small eye movements in the second stage [14]. The frame-to-frame registration step allows averaging of video frames without any distortion. In this way, a sharp mean image can be calculated from a registered video sequence (Fig 2a).

#### Blood vessel segmentation

A gray scale mean image (Fig 2a) that was created by averaging the registered video frames was used as an input for segmentation of blood vessels. The segmentation method utilizes a previously published approach based on matched filtering and support vector machine classification technique [15]. The extracted binary vessel masks (Fig 2b) represent the structure of the blood vessels in each image. These masks allow restricting the further analysis to areas without visible vessels, i.e. in retinal structures with micro capillaries on the background of the image, which are important for delivering the blood to the functional retinal tissues. We restricted the analysis to non-vessels areas, because we wanted to focus only on blood volume modulated intensity changes in retinal tissue (microcapillaries), not influenced by physiological cardiac processes in larger blood vessels itself.

#### Optic disc localization

A binary ONH mask was selected for each mean image in the experimental dataset. The masks were created manually by labelling the ONH borders by an experienced person. The inner part of the contour was morphologically filled and smoothed to obtain binary representation of the ONH area (Fig 2c). The ONH center was calculated as the center of gravity of the closed binary area. Using the morphological operations, a diameter (radius) of the ONH was calculated for each subject.

#### PAA maps calculation

**Fig 3:**
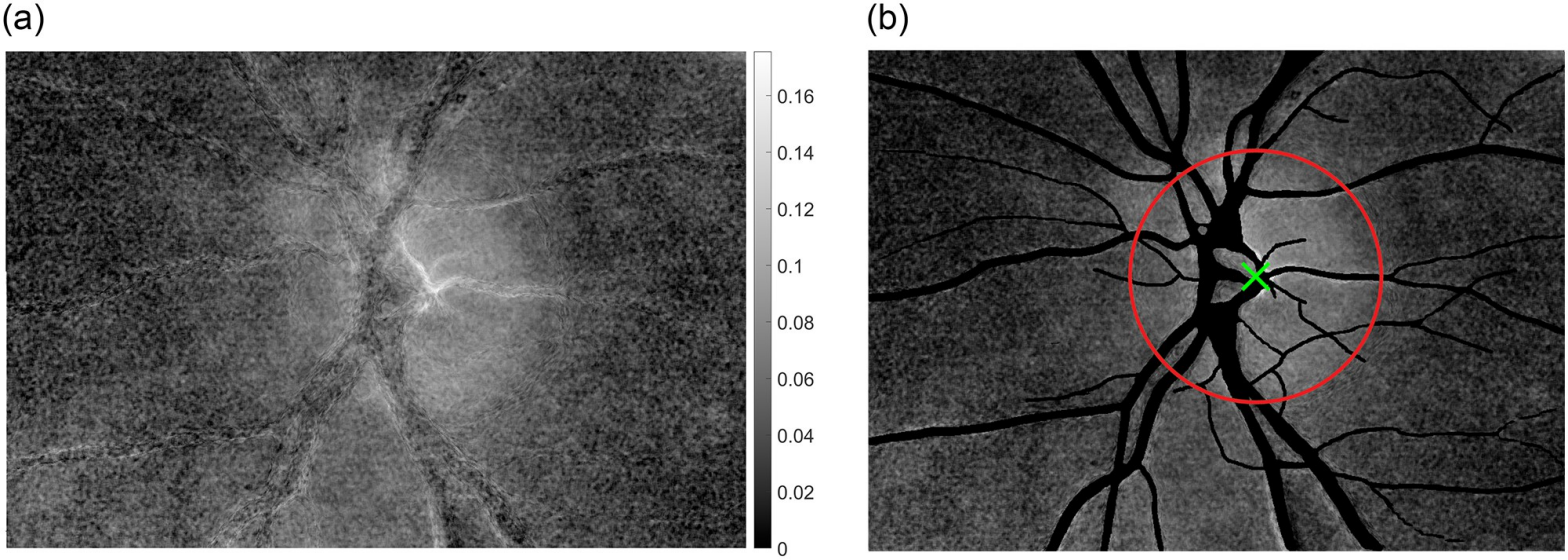
Calculated PAA maps. a) *PAA*(*x*,*y*) spatial distribution, b) *PAA*(*x*,*y*) masked by the blood vessels (black areas). The colorbar shows PAA value. The green cross depicts the center of ONH and the red ring the approximation of the ONH border.

As described previously [11], the pulsatile attenuation amplitude (PAA) can be used to assess the change in light attenuation caused by pulsatile blood volume changes in examined retinal tissue. During each cardiac cycle, increasing blood volume results in higher light absorption in retinal tissue, which causes lower image intensity (and vice versa). Thus, ignoring light scattering, the maximum change of light attenuation *A*_*max*_ during one cardiac cycle (i.e. PAA) can be expressed as follows [11]:

$$PAA=A_{max}=1-\frac{I_{min}}{I_{max}},$$ (1)

where *I*_*min*_ and *I*_*max*_ represent minimum (the highest blood volume) and maximum (the lowest blood volume) image intensity during one heartbeat, respectively [11]. The spatial distribution *PAA*(*x*,*y*) (Fig 3a) can be calculated by applying Eq (1) to each pixel of particular video frames in registered retinal video sequence.

The pixel-wise signal of pulsatile attenuation changes is taken to extract the heartbeat cycles (pulses) for the calculation of *I*_*min*_ and *I*_*max*_. According to the previous work [11], the final *PAA(x*,*y)* maps were obtained by averaging particular *PAA*(*x*,*y*) maps computed from 5 different heartbeat pulses which were manually determined within the sequence. The manual selection enables to utilize only undistorted pulses (without e.g. eye blinks or eye movements during the acquisition). Given the *PAA*(*x*,*y*) spatial distribution, the particular gray values can be obtained from the non-vessels areas (Fig 3b). The obtained values are then proportional to the cardiac cycle-induced changes in blood volume in a retinal tissue without visible blood vessels.

The circular OCT scans provide RNFL thickness values for each eye included in the study dataset. To be able to compare spatially distributed PAA values with the thickness measurement of the RNFL, the mean values of *PAA*(*x*, *y*) and circular RNFL scans were considered for the analysis.

### PAA-RNFL correlation analysis

The main idea to reveal the relation between PAA and RNFL thickness values is to compute the Pearson correlation coefficient *R* between the mean PAA values and mean RNFL thickness. Different areas (evaluating patterns) for PAA calculation are proposed as described below. RNFL thickness naturally decreases with age. Therefore, we performed calculation of the partial correlation coefficient with a control variable, which is an age of particular subjects in the study. It is assumed that controlling the effect of age in the computation of correlation coefficient gives us more accurate results expressing better the true dependence of PAA parameter and RNFL thickness.

#### Blood vessel-free area of the ONH

PAA_ONH_ is the average value calculated from pixels in the area of the whole ONH surface without visible vessels. This is calculated using the vessel mask and the ONH mask. This means that only the vessel-free pixel locations inside the ONH outlined by the binary ONH mask were considered in PAA_ONH_ calculation (Figs 2c and 3b). The PAA_ONH_ calculation was included in order to compare the novel results with our previous results [12], which might help to better understand, interpret and also to extend the PAA fundamentals.

#### ONH circular and semi-circular areas

**Fig 4:**
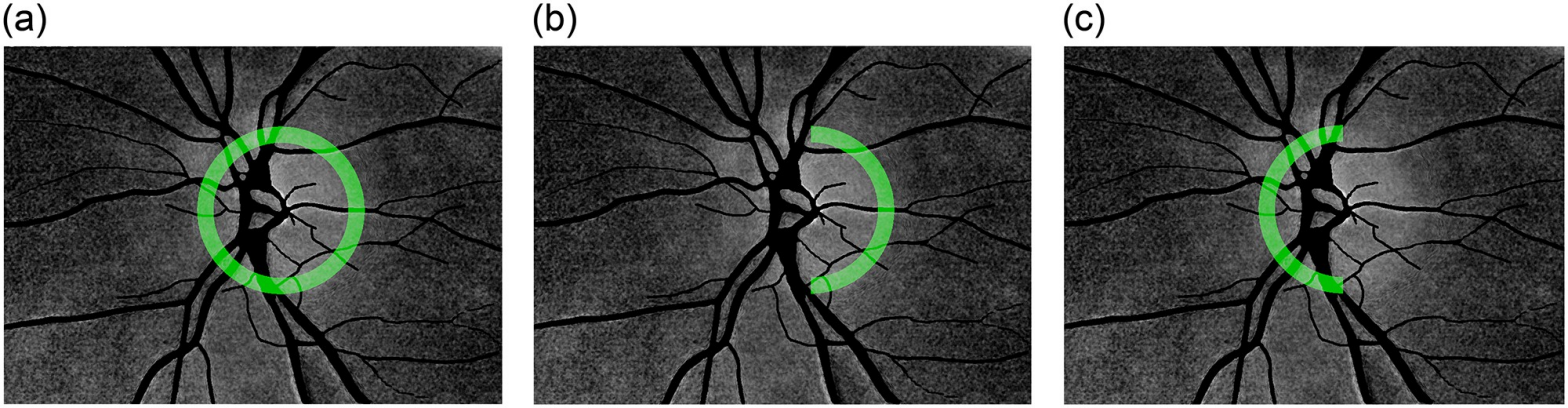
An example of PAA spatial map with masked blood vessels and depiction of the analyzed region in a) 360° circular area (PAA_360_), b) temporal semi-circular area (PAA_temp_), and c) nasal semi-circular area (PAA_nasal_). Blood vessels are excluded from calculation of the PAA parameter.

**Fig 5:**
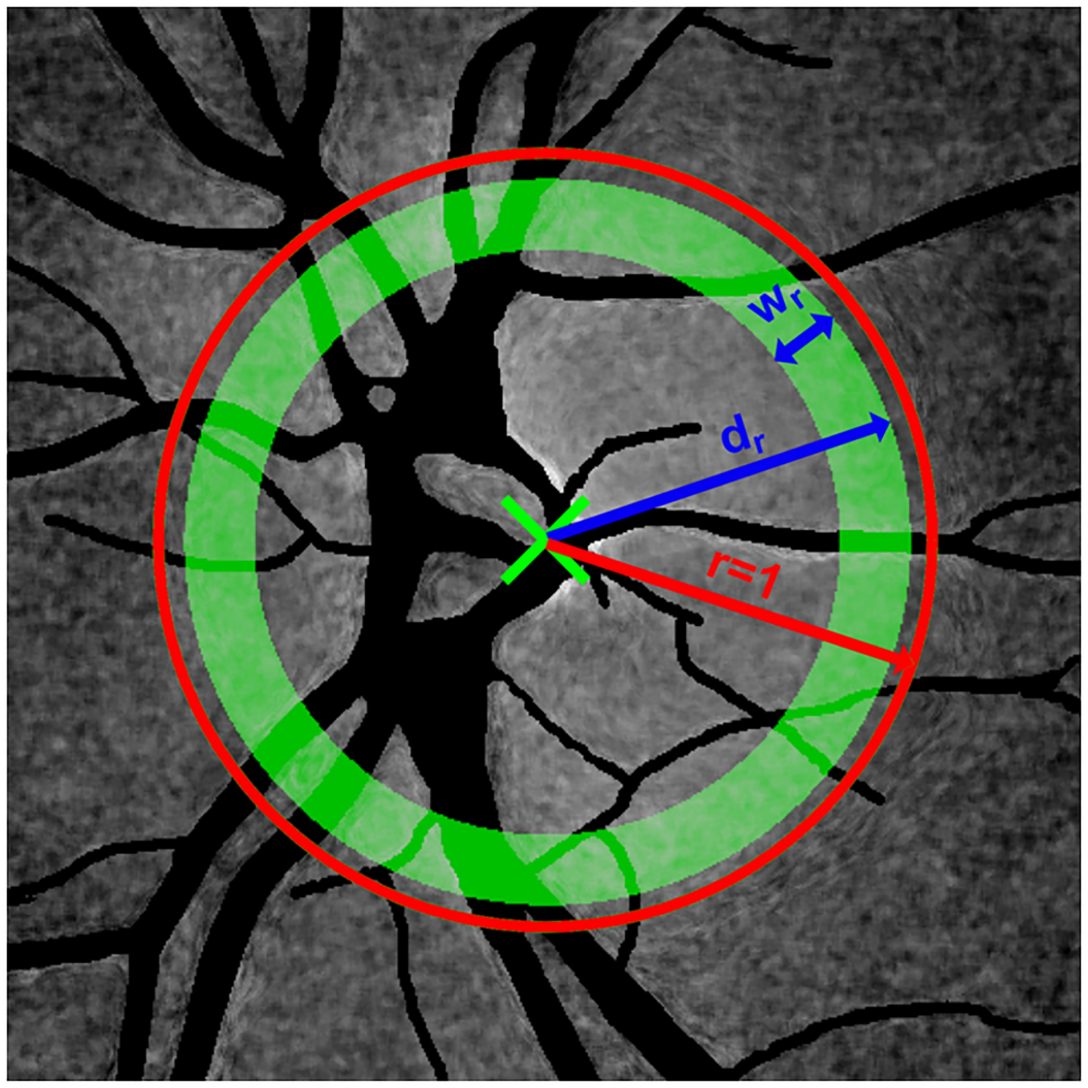
Image showing the annulus pattern in the PAA map and the meaning of the relative parameters *w*_*r*_ (relative width) and *d*_*r*_ (relative distance). The blue color depicts the parameters. The red arrow indicates the ONH radius for a particular eye, normalized to 1 (red circle). Different situations can occur when searching for the optimal values of the proposed parameters. When *d*_*r*_<*r*, the annulus is inside the ONH, while when *d*_*r*_>*r*, the annulus is placed outside the ONH region. Parameter *w*_*r*_ controls the thickness of the annulus, which influences how much of PAA surface will be considered for calculation.

To further examine a statistical relation between PAA spatial maps and RNFL thickness, an analysis in different annulus areas inside and outside the ONH region was conducted. PAA_360_ represents the average PAA value in ONH centered annulus excluding the blood vessels (Fig 4a). PAA_360_ is calculated for different geometrical parameters of the annulus (defined by the annulus width *w* and the annulus distance *d*, with respect to the ONH radius), see graphical explanation in Fig 5. PAA_temp_ represents the temporal semi-circular part of the annulus and PAA_**nasal**_ the nasal semi-circular part of the annulus (Fig 4b and 4c).

The PAA values can be essentially calculated from the PAA map either from a single circle or from the annulus with a certain width *w*>1 pixel (Fig 5). The annulus area was proposed, since we assume that this can be more robust, providing noise-less and more reliable results. The average *PAA*(*x*,*y*) values are then calculated along the binary circular or semi-circular masks as depicted by green color in Fig 4, where the blood vessels were excluded using the vessel masks.

In order to get an optimal position (distance from ONH center) and width of the annulus defined by the parameters *w* and *d* for each particular eye, a full-search optimization approach within a reasonable range constraint for *w* and *d* was utilized. Considering different radius of the ONH *r* within each eye, relative values of *w* and *d* (relative width *w*_*r*_ and relative distance *d*_*r*_) with respect to ONH radius *r* = 1 (red circle in Fig 5) were calculated (i.e. *w*_*r*_ = *w*/*r*, *d*_*r*_ = *d*/*r*). Then, the parameters *w*_*r*_ and *d*_*r*_ were optimized in the range from 0 to 0.25 and from 0 to 1.5, respectively; thus, the absolute values can be determined as *w*_*r*_*·r* and *d*_*r*_*·r*, respectively. It means, that for example, if the *d*_*r*_ = 0.5, then the absolute *d* in pixels is equal to actual half of the ONH radius of a particular eye. The age-controlled correlation coefficient *R* was calculated to find the highest correlation between the mean *PAA(x*,*y*) within the annulus pattern and the mean RNFL thickness, which were counted in the same angular correspondence as with the annulus masks in Fig 4. Thus, 360°, temporal, and nasal areas were mutually compared. *R* was then calculated over the whole dataset (for a particular combination of *w*_*r*_ and *d*_*r*_). In this way, the optimal average values of *w*_*r*_ and *d*_*r*_ that maximize the PAA-RNFL correlation can be found. This allows a proper setup of the PAA-RNFL analysis with respect to each particular eye with anatomically different ONH radius.

## Results

**Fig 6:**
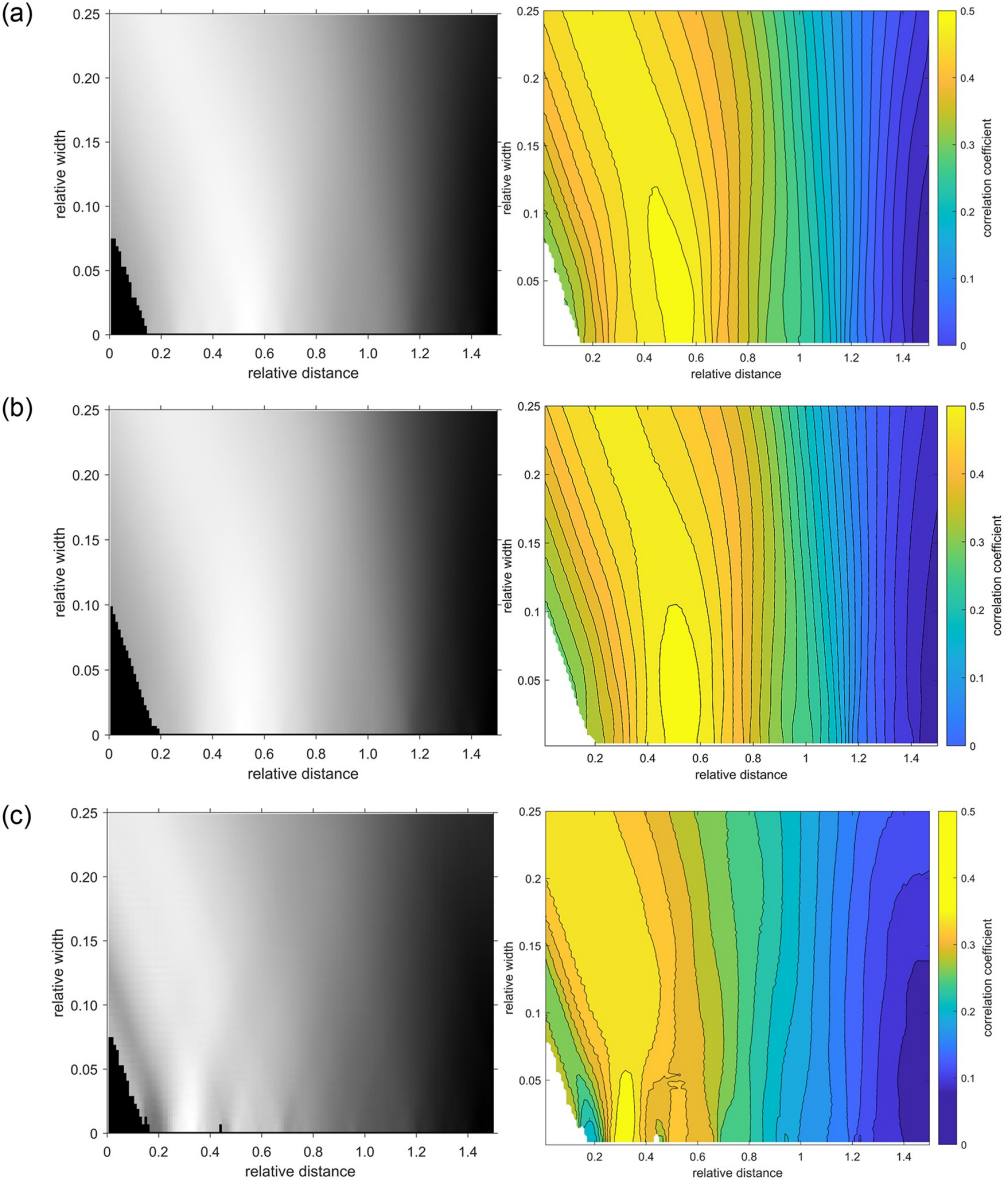
Values of correlation coefficient as a result of full-search optimization for optimal parameter settings. Left: Images showing the result of full-search computation maximizing the average value of *R*; the lightest areas correspond to the highest values of *R*. Right: The values of *R* drowned in isolines as a contour plot. The most yellow color depicts the region with approximately 95% of maximum correlation. Results are shown for a) the 360° area, b) the temporal area, and c) the nasal area.

Fig 6 shows the calculated distribution of the correlation coefficient for 3 different areas (360° circular area, temporal and nasal semi-circular area) in grey values (left) and as a color-coded contour plot with isolines (right) as a function of relative distance *d*_*r*_ and relative width *w*_*r*_. The highest correlation region for *d*_*r*_ is in the range from approx. 0.4 to 0.6 for 360° circular area and temporal semi-circular area (Fig 6a and 6b) and at about 0.3 for the nasal semi-circular area (Fig 6c). The most yellow region represents about 95% of maximum correlation. While the decrease in correlation with deviation from the maximum values is steep for *d*_*r*_ (perpendicular to isolines), it is much shallower for *w*_*r*_ (along isolines). The PAA-RNFL analysis revealed the highest correlation coefficient for the temporal semi-circular area (Fig 6b): *R*_*temp*_ = 0.557 (p<<0.001) with the parameters *d*_*r*_ = 0.510, *w*_*r*_ = 0.004. For the other areas, the analysis showed lower values of correlation. The 360° circular area analysis (Fig 6a) revealed *R*_*360*_ = 0.530 (p<<0.001) with *d*_*r*_ = 0.55 and *w*_*r*_ = 0.008. The nasal semi-circular area analysis (Fig 6c) showed *R*_*nasal*_ = 0.332 (p<<0.001) with *d*_*r*_ = 0.330, *w*_*r*_ = 0.008. For comparison, the correlation coefficient for the entire ONH is *R*_*ONH*_ = 0.459 (p<0.001).

Table 1 summarizes the optimal relative parameter values of the annulus and corresponding maximal values of correlation coefficients. In this table, the absolute values *w* and *d* are also expressed in pixels. This was calculated with the mean value of ONH radius in the dataset, which is equal to 171±15 pixels (computed from all 111 eyes).

**Table 1:** Comparison of estimated optimal parameter values and correlation coefficients.

	Correlation coefficient	Optimal distance *d*_*r*_/*d*	Optimal width *w*_*r*_/*w*
**PAA**_**360**_	*R*_*360*_ = 0.530	*d*_*r*_ = 0.55 / 94.1 px	*w*_*r*_ = 0.008 / 1.4 px
**PAA**_**temp**_	*R*_*temp*_ = 0.557	*d*_*r*_ = 0.510 / 87.2 px	*w*_*r*_ = 0.004 / 1.0 px
**PAA**_**nasal**_	*R*_*nasal*_ = 0.332	*d*_*r*_ = 0.330 / 56.4 px	*w*_*r*_ = 0.008 / 1.4 px
**PAA**_**ONH**_	*R*_*ONH*_ = 0.459	NA	NA

All correlations were statistically significant at the significance level of p<0.001. The pixel values of particular parameters were computed using the average ONH radius *r* = 171 ± 15 pixels.

**Fig 7:**
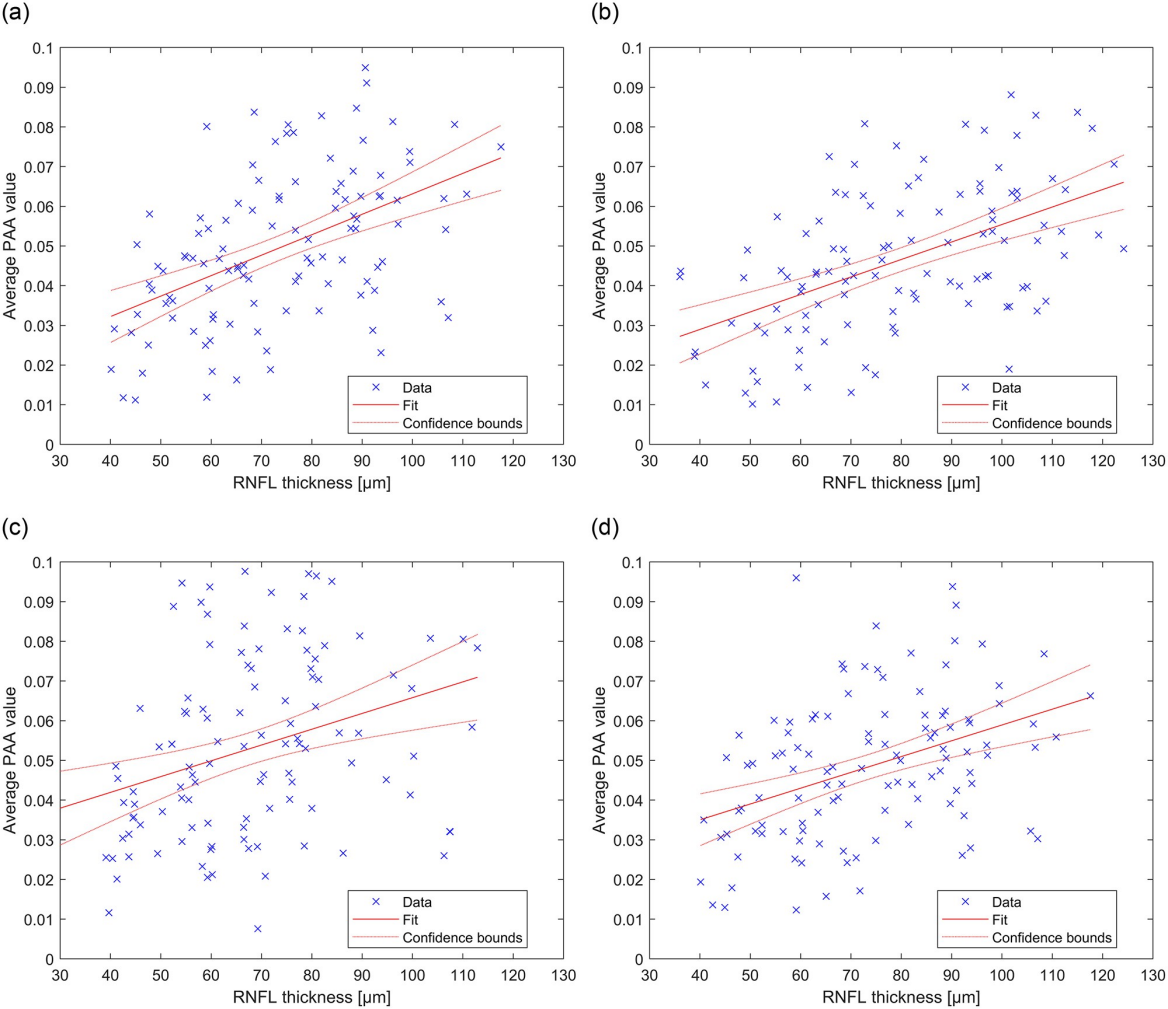
Statistical relation between RNFL thickness and PAA values for each eye in the dataset. Values of PAA were obtained with the optimal parameters setting (Table 1) maximizing the correlation coefficient. The graphs show particular relations between RNFL thickness and a) PAA_360_ (*R* = 0.530), b) PAA_temp_ (*R* = 0.557), c) PAA_nasal_ (*R* = 0.332), d) PAA_ONH_ (*R* = 0.459).

Utilizing the optimal parameter settings *d* and *w* in its absolute values (Table 1), the scatter plots at Fig 7 show statistical dependence between PAA and RNFL thickness for 4 evaluating patterns (i.e. PAA_360_, PAA_temp,_ PAA_nasal,_ PAA_ONH_) together with regression lines and corresponding confidence bounds. The highest correlation obtained for PAA_temp_ can be clearly observed from the graph in Fig 7b, while the points that belong to the nasal region (Fig 7c) are much more scattered.

**Fig 8:**
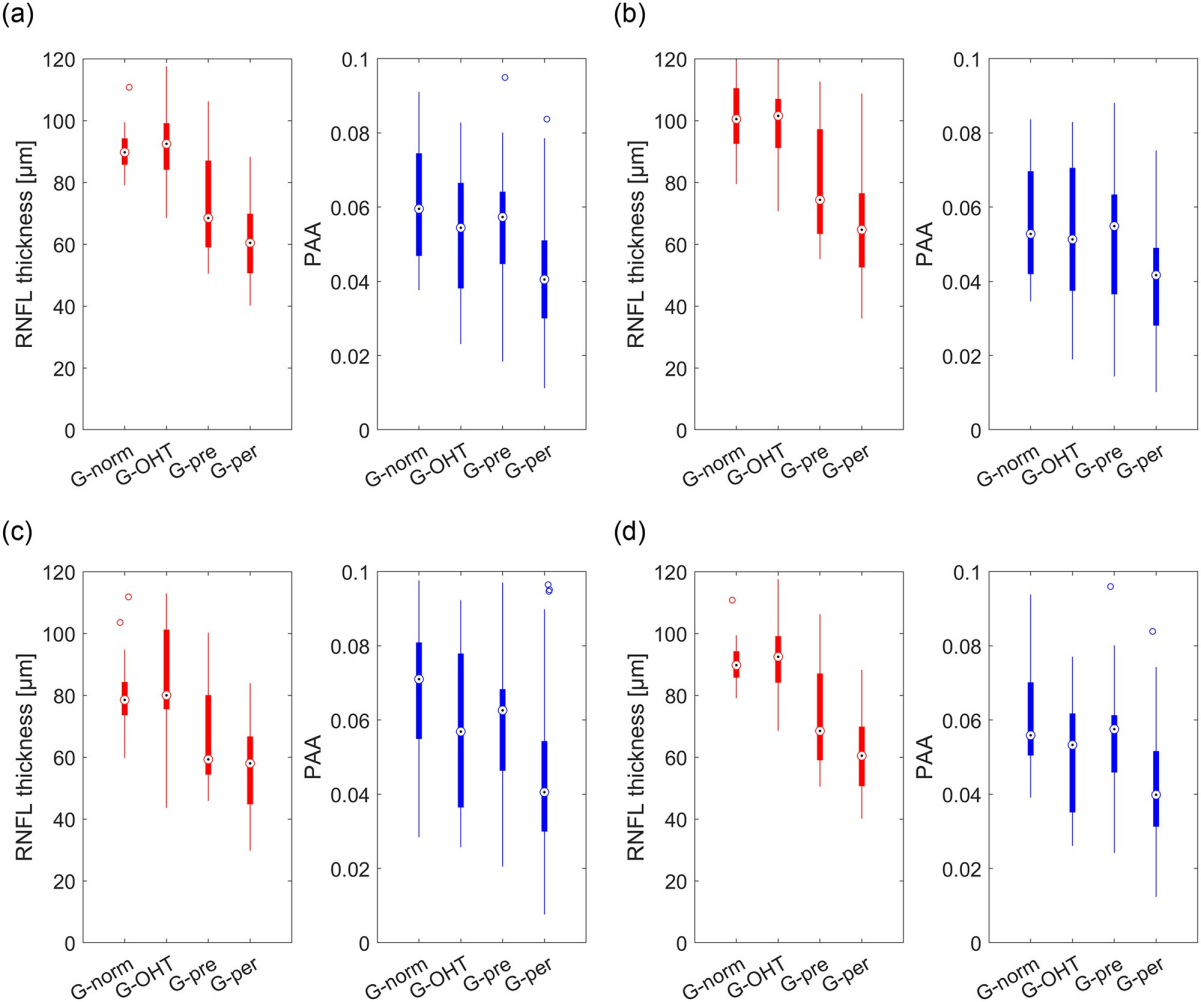
Boxplots showing distribution of the values of mean RNFL thickness (red color) and PAA (blue color) in individual glaucoma groups (G_norm_-G_per_) for all calculated areas of interest. a) 360° circular area, b) temporal semi-circular area, c) nasal semi-circular area, and d) entire ONH area. The values were calculated with the optimal parameter setting as shown in Table 1.

Finally, Fig 8 shows results for RNFL thickness and PAA in four subject groups (G_norm_, G_OHT,_ G_pre_, G_per_) for all calculated areas of interest. As with the result for RNFL thickness, there is a decrease in PAA value with increasing glaucoma severity. However, while for the RNFL thickness a decrease is visible from G_OHT_ to G_pre_, for PAA a clear decrease is between G_pre_ and G_per_.

## Discussion

The results of correlation analysis conducted in a whole ONH area (*R*_*ONH*_*)* agrees with the methodology reported previously [12]. Table 1 shows the absolute and relative values of parameters *d* and *w* calculated using the mean value of ONH radius *r* = 171±15 pixels. Although, the analysis is matched to the actual ONH radius for each particular eye, these (average) values of *d* and *w* indicate that it is useful to utilize a thin evaluating pattern placed in the region inside the ONH (*d* < *radius of ONH; d*_*r*_ *< 1*) to achieve the highest correlation between PAA and RNFL thickness. The relative value *d*_*r*_ = 0.55 for PAA_*360*_ indicates that the highest correlation is obtained in the distance corresponding to about the half of the ONH radius. In Fig 6a, shallower appearance for *w*_*r*_ means that the parameter *w*_*r*_ can have a relatively high range (approx. up to *w* = 21.3 pixels) and it is not that critical for maintaining an adequate correlation. Nevertheless, this result shows that the PAA evaluated inside the ONH region (particularly close to the neuroretinal rim—the area between the optic cup and the ONH border) has the highest correlation with the mean value of the RNFL thickness. The highest correlation was achieved in the temporal area (PAA_temp_) which is likely due to the anatomical fact that this region contains the papillomacular bundle located in the temporal ONH with the highest density of small retinal ganglion cells that require a high volume of blood supply because of the increased mitochondrial metabolic demands. This finding is also consistent with results published in recent papers focused on clinical application of OCTA. For example, Jia et al. [16] showed flow index and vessel density reduction for three glaucoma subjects in temporal regions of the ONH. Lévêque et al. [3] found statistically significant correlation between the thickness of the RNFL and blood vessel density in both, the whole and the temporal part of the ONH, for advanced glaucoma cases. The density and flow index of blood vessels provided by OCTA means that in the case of their reduction, the blood volume flowing through these tissues is reduced. The results are also consistent with the assumption that the correlation is expected to be lower in the nasal area (PAA_nasal_), due to the lower occurrence of nerve fibers. The graphs shown in Fig 6b and 6c revealed, similarly as in case of the entire annulus area (360°), that there is a rather high range of *d*_*r*_ and *w*_*r*_ values possible for maintaining the correlation above the 95% of the maximum for temporal semi-circular pattern. On the contrary, the nasal region shows a shallower area where the correlation can be maintained significantly high (>95%).

The study also showed that higher correlation can be expected for narrower annulus. This is evidenced by higher values of the correlation coefficient, compared to the whole surface ONH analysis according to the methodology presented earlier in [12] (see PAA_ONH_ results in Table 1). The suitability of placing the annulus inside the ONH region may also indicate that the optimal position corresponds anatomically to the inner border of the optic cup. The findings revealed that it is probably useful to focus on analyzing the PAA near the center of the ONH, in the area of the optic cup or the neuroretinal rim, which are very important diagnostic structures (especially in glaucoma diseases) [17, 18]. These findings are also in correlation with the study published by Jonas et al. [18]. They presented wide ONH morphometric data showing a typical range of neuroretinal rim width (relatively 0.45–0.60). This corresponds to our findings represented by *d*_*r*_ parameter.

The largest variation in RNFL thickness among different glaucoma groups was observed for 360°-analysis and in the temporal semi-circular region (Fig 8). This is in correlation with a prior anatomical assumption that the density of nerve fibers is much higher in the temporal part compared to the nasal part. The median value of RNFL thickness (shown by red color in boxplot graphs in Fig 8) decreases clearly for the pre-perimetric (G_pre_) and the perimetric (G_per_) glaucoma group, compared to the normal (G_norm_) group. Similarly, PAA values decrease according to decrease of RNFL thickness. For PAA, the decrease is mainly visible for the perimetric (G_per_) glaucoma group. This finding can be due to the fact that in pre-perimetric glaucoma (G_pre_) with no visual field damage the blood supply of the retinal tissue is still sufficiently preserved. Then, the retinal tissue with insignificant structural damage and preserved blood circulation does not have a substantial effect, which can be reflected in PAA analysis. These findings are consistent with OCTA studies published by Mangouritsas [19] or Kim [20]. In [19, 20], the peripapillary microvasculature at the areas with RNFL defect was found similar in density for normal and pre-perimetric glaucoma eyes, but in perimetric glaucoma, a significant decrease in vessel density was observed. Our findings may suggest (similar to studies [19, 20]) that the microvascular dysfunction in the retina is a secondary change to RNFL degeneration in the pathogenesis of glaucoma, preceded by the ganglion cells degeneration and axonal loss. However, more investigation is still needed with larger dataset and possibly improvement and automatization of PAA estimation from the original video frames to prove this assumption. Before transition of this research into the clinical space, more measurement and deeper analysis of the PAA ability to reflect changes in peripapillary blood vessel density and consequently blood supply in glaucoma have to be performed. Furthermore, it has to keep in mind that the blood supply in the ONH area is not only affected by diseases like glaucoma, diabetes and stenosis of the carotid arteries, but also by other parameters like blood pressure and body mass index.

## Conclusion

The proposed study confirmed that the photoplethysmographic principle can be used to analyze changes in the blood supply of the retinal tissue in ONH and peripapillary area. This can potentially be used to assess the progression of changes in blood supply of the ONH connected to glaucoma and other diseases. Using correlation analysis, we determined the most suitable position for measuring the PAA parameter, with respect to the evaluation of variations in RNFL thickness. The results revealed the highest correlation between PAA and RNFL when measured in the temporal areas and inside the ONH region. The results were also compared with the methodology published earlier in [12]. The advantage of the proposed approach using an innovative video ophthalmoscope opens the possibility of binocular measurement in the future. This might allow simultaneous comparison of clinically relevant blood flow parameters between the two eyes, even fast dynamic parameters like rise time of pulses and possible delay between both eyes. This type of analysis brings new possibilities and insights for the diagnosis of glaucoma as well as other diseases that correlate with reduced blood flow of the retinal tissue and subsequent structural changes. Besides technical improvements, ongoing research will further focus on deeper comparison of PAA potential in connection with the use of the proposed approach to determine clinically useful parameters of retinal tissue perfusion. Further studies should also evaluate the proposed principle compared to clinically established approaches such as OCTA or LSF.

## References

[1] Sathyan P, Shilpa A. Optical Coherence Tomography in Glaucoma. *Journal of Current Glaucoma Practice*. 2012;6(1):1. doi: 10.5005/jp-journals-10008-1099 27990063 PMC5159451

[2] Rabiolo A, Carnevali A, Bandello F, et al. Optical coherence tomography angiography: evolution or revolution? *Expert Review of Ophthalmology*. 2016;11(4):43–245.

[3] Lévêque P-M, Zéboulon P, Brasnu E, et al. Optic disc vascularization in glaucoma: value of spectral-domain optical coherence tomography angiography. *Journal of Ophthalmology*. 2016; Article ID 6956717:1–9. doi: 10.1155/2016/6956717 26998352 PMC4779818

[4] Richter GM, Sylvester B, Chu Z, et al. Peripapillary microvasculature in the retinal nerve fiber layer in glaucoma by optical coherence tomography angiography: focal structural and functional correlations and diagnostic performance. *Clin Ophthalmol*. 2018;12:2285–2296. doi: 10.2147/OPTH.S179816 30510397 PMC6231432

[5] Li Z, Xu Z, Liu Q, et al. Comparisons of retinal vessel density and glaucomatous parameters in optical coherence tomography angiography. *PLoS ONE*. 2020;15(6):e0234816. doi: 10.1371/journal.pone.0234816 32584833 PMC7316331

[6] Zha Y, Chen J, Liu S, et al. Vessel density and structural measurements in primary angle-closure suspect glaucoma using optical coherence tomography angiography. *BioMed Research International*. 2020; Article ID 7526185:1:6. doi: 10.1155/2020/7526185 34258258 PMC8243897

[7] Khayrallah O, Mahjoub A, Abdesslam N-B, et al. Optical coherence tomography angiography vessel density parameters in primary open-angle glaucoma. *Annals of Medicine and Surgery*. 2021;69. doi: 10.1016/j.amsu.2021.102671 34408871 PMC8361292

[8] Konishi N, Tokimoto Y, Kohra K, et al. New laser speckle flowgraphy system using CCD camera. *OPT REV*. 2002;9:163–169.

[9] Yokoyama Y, Aizawa N, Chiba N, et al. Significant correlations between optic nerve head microcirculation and visual field defects and nerve fiber layer loss in glaucoma patients with myopic glaucomatous disk. *Clin Ophthalmol*. 2011;5:1721–1727 doi: 10.2147/OPTH.S23204 22205831 PMC3245193

[10] Shiga Y, Kunikata H, Aizawa N, et al. Optic nerve head blood flow, as measured by laser speckle flowgraphy, is significantly reduced in preperimetric glaucoma. *Current Eye Research*. 2016;41(11):1447–1453. doi: 10.3109/02713683.2015.1127974 27159148

[11] Tornow RP, Odstrcilik J, Kolar R. Time-resolved quantitative inter-eye comparison of cardiac cycle-induced blood volume changes in the human retina. *Biomed Opt Express*. 2018;9(12):6237–6254. doi: 10.1364/BOE.9.006237 31065425 PMC6490987

[12] Tornow RP, Kolar R, Odstrcilik J, et al. Imaging video plethysmography shows reduced signal amplitude in glaucoma patients in the area of the microvascular tissue of the optic nerve head. *Graefes Arch Clin Exp Ophthalmol*. 2021;259:483–494. doi: 10.1007/s00417-020-04934-y 32960321 PMC7843566

[13] Kolar R, Odstrcilik J, Tornow RP. Photoplethysmographic analysis of retinal videodata based on the Fourier domain approach. *Biomed*. *Opt*. *Express*. 2021;12:7405–7421. doi: 10.1364/BOE.441451 35003842 PMC8713668

[14] Kolar R, Tornow RP, Odstrcilik J, et al. Registration of retinal sequences from new video-ophthalmoscopic camera. *BioMed Eng OnLine*. 2016;15(57). doi: 10.1186/s12938-016-0191-0 27206477 PMC4875736

[15] Odstrcilik J, Kolar R, Budai A, et al. Retinal vessel segmentation by improved matched filtering: evaluation on a new high-resolution fundus image database. *IET Image Processing*. 2013;7:373–383.

[16] Jia Y, Morrison JC, Tokayer J, et al. Quantitative OCT angiography of optic nerve head blood flow. *Biomed*. *Opt*. *Express*. 2012;3:3127–3137. doi: 10.1364/BOE.3.003127 23243564 PMC3521313

[17] Kumar JRH, Seelamantula CS, Kamath YS, et al. Rim-to-disc ratio outperforms cup-to-disc ratio for glaucoma prescreening. *Sci Rep*. 2019;9:7099. doi: 10.1038/s41598-019-43385-2 31068608 PMC6506519

[18] Jonas JB, Gusek GC, Naumann GO. Optic disc, cup and neuroretinal rim size, configuration and correlations in normal eyes. *Invest*. *Ophthalmol*. *Vis*. *Sci*. 1988;29(7):1151–1158. 3417404

[19] Mangouritsas G, Koutropoulou N, Ragkousis A et al. Peripapillary vessel density in unilateral preperimetric glaucoma. *Clin Ophthalmol*. 2019;13:2511–2519. doi: 10.2147/OPTH.S224757 31997876 PMC6917599

[20] Si Kim, Eun Lee, Chul Han Jong et al. Comparison of peripapillary vessel density between preperimetric and perimetric glaucoma evaluated by OCT-angiography. *PLOS ONE*. 2017;12: e0184297. doi: 10.1371/journal.pone.0184297 28859176 PMC5578657

